# Systemic Lupus Erythematosus (SLE) Induced by ASIA Syndrome After the Aesthetic Medicine Procedures—A Case Report

**DOI:** 10.3390/jcm14010119

**Published:** 2024-12-28

**Authors:** Michalina Knapik, Agnieszka Owczarczyk-Saczonek, Łukasz Jaśkiewicz, Jakub Kuna, Grzegorz Chmielewski, Magdalena Krajewska-Włodarczyk

**Affiliations:** 1 Department of Rheumatology, School of Medicine, Collegium Medicum, University of Warmia and Mazury in Olsztyn, 10-719 Olsztyn, Poland; kuna.jakub@wp.pl (J.K.); gchmielewski.gc@gmail.com (G.C.); 2 Department of Dermatology, Sexually Transmitted Diseases and Clinical Immunology, School of Medicine, Collegium Medicum, University of Warmia and Mazury in Olsztyn, 10-719 Olsztyn, Poland; aganek@wp.pl; 3 Department of Human Physiology and Pathophysiology, School of Medicine, Collegium Medicum, University of Warmia and Mazury in Olsztyn, 10-719 Olsztyn, Poland; lukasz.jaskiewicz@uwm.edu.pl

**Keywords:** autoimmune syndrome induced by adjuvants, systemic lupus erythematosus, aesthetic medicine procedures, case report, ASIA, SLE, delayed inflammatory reaction, DIR

## Abstract

**Introduction:** The autoimmune/inflammatory syndrome induced by adjuvants (ASIA) is a rare condition caused by an immune response associated with over-reactivity of the immune system, triggered by adjuvants. The most common adjuvants are aluminium salts but can also be bioimplants or infectious agents. It may lead to the development of various autoimmunologic diseases. **Case Report:** In the following article, we present the case of a 26-year-old woman who developed SLE likely induced by ASIA syndrome after the aesthetic medicine procedures. The patient was admitted because of arthralgia and fever. She also presented with a butterfly-shaped erythema on her face and erythematous and infiltrative skin lesions on the posterior surface of the thighs and buttocks. We performed numerous diagnostic tests, including laboratory tests, immunological tests, imaging diagnostics such as chest X-ray and USG of the abdomen and joints, and the biopsy of the skin lesion on the left thigh. The results of the diagnostic process led us to diagnose SLE. The patient fulfilled the ACR/EULAR 2019 classification criteria of the SLE. Laboratory results also led to the diagnosis of autoimmune haemolytic anaemia. Due to exposure to numerous adjuvants like tattoo ink, hyaluronic acid, and piercing and the development of the delayed inflammatory reaction (DIR) to hyaluronic acid (HAF), the patient also fulfilled the criteria of ASIA. In the treatment process we applied antibiotics, non-steroidal anti-inflammatory drugs (NSAIDs), steroids, hydroxychloroquine, and cyclosporine. The treatment resulted in an improvement in the general condition, resolution of swelling and joint pain, and improvement in skin lesions. **Conclusions:** ASIA syndrome after bioimplantation is still underdiagnosed, probably due to ignorance or diagnostic difficulties, as symptoms are uncharacteristic and there is no immunological marker for this syndrome. In addition, as in the presented case, it can develop several years after the procedure, and it is difficult for both patient and physician to become aware of the connection. Early diagnosis requires a multidisciplinary approach and may require immunosuppressive treatment in specific cases.

## 1. Introduction

The autoimmune/inflammatory syndrome induced by adjuvants (ASIA) is an immune response associated with over-reactivity of the immune system, triggered by adjuvants [1]. The classic adjuvants are aluminium salts, which are used in vaccines to increase the immunogenicity of the vaccines, both innate and adaptive immune responses to the administered bacterial or viral antigen [1,2]. The role of the adjuvant is to protect the antigens from degradation, thus giving them the opportunity for prolonged exposure to antigen-presenting cells (APCs), resulting in greater immunogenicity and thus vaccine efficacy [1,2]. In addition, the adjuvant drives pattern recognition receptor (PRR) activation of the innate immune system, leading to APC stimulation. Adjuvants can also be infectious agents, bioimplants (silicone, acrylamides, hyaluronic acid (HA), methacrylate compounds, metal joint implants) [3,4,5,6]. Immediately after the biomaterial is implanted into the tissue, it becomes surrounded by phagocytes (mainly macrophages of the pro-inflammatory M1 subtype, then polarising into the anti-inflammatory M2 subtype), as a normal, physiological response in the healing process [4,7]. Under the influence of stimulants (e.g., bacterial lipopolysaccharides, IFN), macrophages may convert back to the pro-inflammatory M1 phenotype [8].

Genetic predisposition plays an important role in this reaction, particularly the presence of genes predisposing to the development of autoimmune diseases (HLA-DRB1*01 or HLA-DRB4). Adjuvant molecules bind to toll-like receptors (TLRs) on APC cells, activating the inflammasome or directly activating macrophages (or other phagocytic cells such as neutrophils), natural killer (NK) lymphocytes, or innate lymphoid cells. In addition, adjuvants enhance the adaptive response by promoting the interaction of dendritic cells with T cells and increasing antigen uptake by APC cells [9]. This leads to the production of non-specific autoantibodies and loss of immune tolerance to one’s own antigens [10].

The diagnostic criteria for ASIA syndrome were introduced by Shoenfeld in 2011. There are ten major criteria and four minor criteria. The detailed characteristics of the criteria are presented in Table 1. To be diagnosed with ASIA syndrome, the patient must fulfil at least two major or one major and two minor criteria.

So far, cases of SLE have been described in the literature as a consequence of ASIA, induced by HBV and HPV vaccination, and following booster doses of diphtheria, tetanus, and pertussis vaccines [11,12]. The concept has emerged that aluminium adjuvant may be responsible for the induction of SLE by promoting cell death, allowing nuclear antigens to move freely, and potentially activating toll-like receptors (TLRs). Furthermore, aluminium-induced IL-6 production induces phenomena leading to the production of autoantibodies, facilitating the development of SLE [11]. On the other hand, patients with SLE are more likely to be predisposed to developing ASIA [9].

We present the case of a patient in whom exposure to adjuvants (hyaluronic acid filler [HAF], tattoos, piercings) provoked ASIA syndrome, likely resulting in the development of autoimmune disorders in the form of SLE and autoinflammatory disorders in the form of Sweet’s syndrome. Although it cannot be ruled out that SLE did not develop spontaneously, the coincidence of ASIA and SLE symptoms implies that there is a connection between these diseases in the presented case.

## 2. Case Report

A 26-year-old woman was admitted to the Department of Rheumatology because of persistent pain and swelling in the joints of the hands, knees, and ankles for about a month, pain in the region of the anterior chest wall when breathing deeply and changing body position, sub-febrile states, and fevers up to 38 °C. The joint pain was also accompanied by morning stiffness lasting up to several hours, causing difficulty in moving around. The patient was only symptomatically using NSAIDs with mediocre results. In addition, the woman had no chronic illnesses, denied allergies, and was not taking any permanent medication. In her history, she reported drinking alcohol once every few months, and in the past she had smoked 5 cigarettes a day (1.75 packs) for 7 years and quit 5 years ago. Her family history stated that her mother suffers from RA (rheumatoid arthritis).

**Figure 1:**
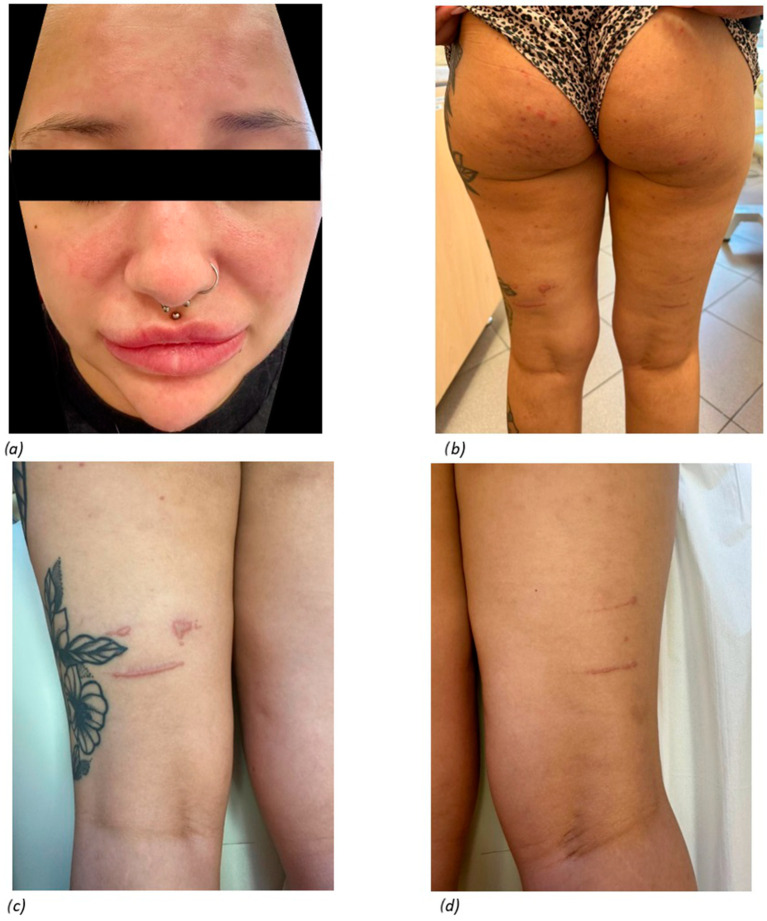
Skin lesions of the patient on admission to hospital: (**a**) erythema and oedema on the face; (**b**) lesions on the posterior surface of the thighs and buttocks; (**c**) lesions on the posterior surface of the left thigh; (**d**) lesions on the posterior surface of the right thigh.

On physical examination on admission, the facial skin showed a fairly well-demarcated, not very intense erythema located on the cheeks, dorsum of the nose, and chin, possibly corresponding to “malar rash” and a large swelling of the mouth and chin area. In addition, erythematous and infiltrative skin lesions with vesicle formation on the surface of the lesions were observed on the posterior surface of the thighs and buttocks (Figure 1). With regard to the musculoskeletal system, painful swelling was present in the proximal interphalangeal and metacarpophalangeal joints of both hands, knee joints, and ankle joints. Moreover, no abnormalities were found on physical examination.

Laboratory abnormalities included elevated inflammatory parameters (CRP 29.2 mg/L, ESR 75 mm/h), leukopenia (2900/μL) with lymphopenia (270/μL), normocytic anaemia (HGB 9.2 g/dL, MCV 90.4 fL), elevated triglycerides (303 mg/dL), D-dimer (3.98 μg/mL), and ferritin (368 ng/mL), and direct antiglobulin test (+). Infections were excluded: anti-HCV, HBS Ag, HIV, and VDRL—negative, antibodies against *Chlamydia trachomatis* in IgM and IgG classes—negative. There were no abnormalities in urinalysis, and GFR and creatinine values remained within the normal range.

**Figure 2:**
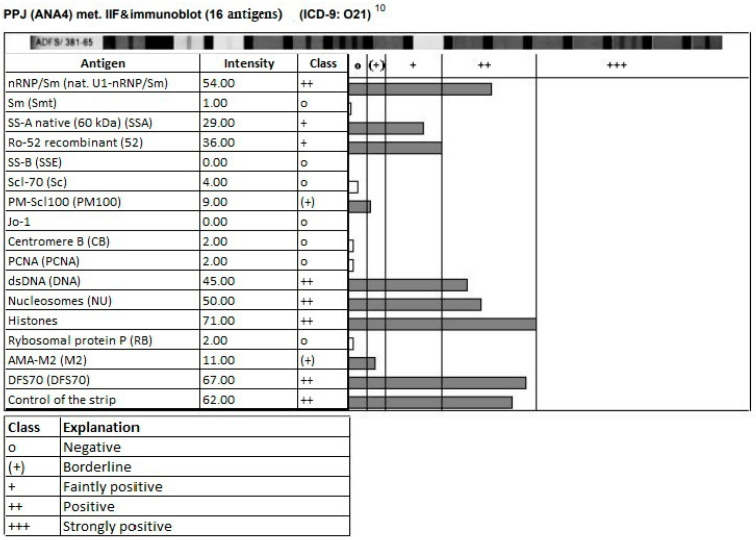
Graphical representation of immunological diagnostic results.

Immunological diagnostics showed anti-CCP < 8 IU/mL, RF 62 IU/mL, and ANA in a titer of 1:5120 granular, 1:320 cytoplasmic, and homogeneous 1:5120, anti-U1RNP++, anti-Ro-52+, anti-SS-A+, anti-dsDNA++, anti-nucleosome antibodies++, and anti-histone antibodies++ (Figure 2); reduced levels of complement components C3 (0.49 g/L) and C4 (0.03 g/L); anti-cardiolipin antibodies negative in both IgM and IgG classes; anti β2-GP1 antibodies positive in IgM class and negative in IgG class; and lupus anticoagulant negative.

Chest X-ray showed no abnormalities, and abdominal ultrasound showed an enlarged spleen 9 mm above normal in the longitudinal dimension. On echocardiography, cardiac morphology and function were normal, with no fluid in the pericardial sac. In order to exclude pulmonary embolism, a chest CT scan (HR and angio-CT) was performed, which, apart from minor post-inflammatory changes, did not show any significant pathological changes. Joint ultrasound described synovial effusion and swelling in the MCP and PIP joints of both hands, synovial effusion and thickening in the knee joints, and effusion in the ankle and tarsal joints.

During hospitalisation, the patient developed symptoms resembling an anaphylactic reaction on two occasions: swelling of the face, neck, lips, and tongue; numbness of the lips and tongue. The first time was also accompanied by nausea and vomiting of food contents. Reactions to diclofenac were initially suspected, and the drug was discontinued, but after the second episode, which could not be associated with any obvious external factor, it was decided to consult a dermatologist. The consultation was also prompted by skin lesions observed on admission of the patient. During the consultation, the suspicion was raised that the recurrent facial swelling might be the result of a delayed inflammatory reaction (DIR) to a hyaluronic acid (HA) preparation injected into the patient’s facial area in the past, and that the overall clinical presentation might be consistent with ASIA syndrome. The dermatologists’ suspicion of this syndrome was prompted by the patient’s symptoms in combination with the numerous risk factors she presented, such as extensive tattoos on her upper and lower extremities, piercings on her upper lip, nose, and nipples, and multiple hyaluronic acid injections on her lips and chin. The woman started tattooing in 2019 and had her last tattoo in 2023. Piercing took place around 2021/2022. She had been undergoing regular (every 0.5–1 year) lip and chin injections with an unregistered hyaluronic acid preparation (Ejal 40) since around 2017. Her last injection was performed around January/February 2024. Immediately after the aforementioned treatments, the patient did not observe any distressing symptoms. During the consultation, a specimen was also taken from the lesion on the posterior surface of the left thigh for histopathological examination. The microscopic image showed foci of hyperkeratosis and parakeratosis, intraepidermal blisters, abundant inflammatory infiltrates in the dermis composed mainly of neutrophils, features of leucocytoclasia, and small foci of necrosis. This picture was most consistent with Sweet’s syndrome. Furthermore, in the additional tests suggested by the dermatologist, a slight increase in the percentage of alpha-2-globulins (12.23%) in the proteinogram was found.

As part of the treatment, the patient was offered the removal of the piercings and the administration of triamcinolone to the mouth and chin area to suppress local inflammation, but the patient did not agree.

**Figure 3:**
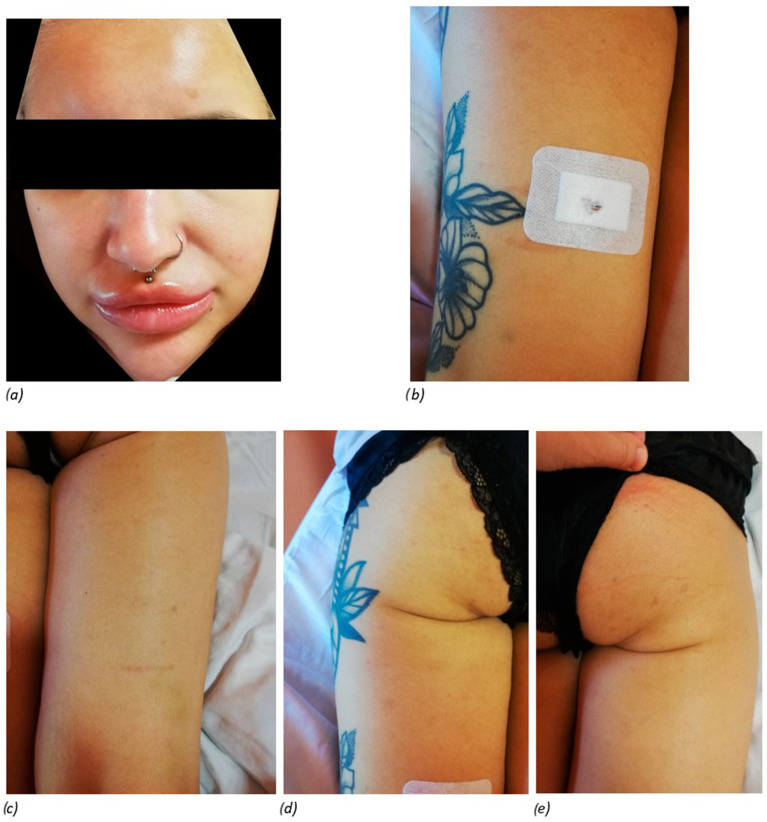
Skin lesions in the patient after two weeks of hospitalisation; (**a**) face; (**b**) left thigh; (**c**) right thigh; (**d**) left buttock; (**e**) right buttock.

The treatment initially included empirical antibiotic therapy with ceftriaxone, followed by pulses of methylprednisolone at a total dose of 3 g, i.v., then oral prednisone at 30 mg/day, hydroxychloroquine at 2 × 200 mg/day, and cyclosporine at 3 mg/kg (2 × 100 mg/day). Analgesic treatment with NSAIDs was also used. Cyclosporine was suggested by the dermatologist as a supportive treatment of the delayed inflammatory reaction and ASIA, as it can reduce the macrophage activity. The treatment resulted in an improvement in the general condition, resolution of swelling and joint pain, and improvement in skin lesions (Figure 3).

On the basis of the ACR/EULAR 2019 classification criteria, the patient was diagnosed with systemic lupus erythematosus (27 points). SLEDAI-2K—12 points (clinical 8 points)—moderate form. Laboratory results also led to the diagnosis of autoimmune haemolytic anaemia. The patient also fulfilled the criteria of ASIA (Table 1). Although Sjogren’s syndrome is a common comorbidity with SLE, our patient did not report any mouth or eye dryness, so we did not perform the diagnostic procedures required to diagnose the Sjogren syndrome in this patient.

The patient was discharged home in a state of general improvement with recommendations to continue treatment with hydroxychloroquine (200 mg 2 × 1 tabs), cyclosporine (100 mg 2 × 1 tabs), and prednisone (30 mg/day) and further follow-up in the rheumatology and dermatology clinics.

## 3. Discussion

In the patient, adjuvants (HAF [hyaluronic acid filler], tattoos, piercings) should be considered as causative factors of ASIA, with the consequent development of SLE.

**Figure 4:**
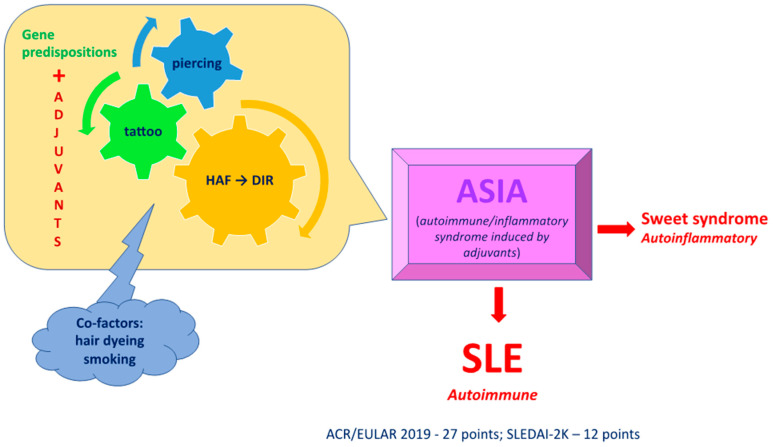
Pathomechanism of the development of the disorder presented in the patient. Exposure to adjuvants (HAF, tattoos, piercings) provoked ASIA syndrome, resulting in the development of autoimmune disorders in the form of SLE and autoinflammatory disorders in the form of Sweet’s syndrome. (HAF—hyaluronic acid filler; DIR—delayed inflammatory reaction).

The HAF (hyaluronic acid filler) administered in the patient provoked DIR (delayed inflammatory reaction), the symptom of which was increased tissue swelling at the site of its implantation (lips, chin). This is a reaction that occurs weeks, months, or years after implantation of the material, with an incidence of 0.5% over 5 years after HAF administration. The triggering factors are conditions leading to a local or systemic increase in the concentration of IFN, a factor that provokes the polarisation of macrophages surrounding the filler towards an M1 inflammatory-type phenotype [4,13,14]. Clinical manifestations are recurrent episodes of localised solid oedema with erythema and tenderness or in the form of subcutaneous nodules of the HAF (hyaluronic acid filler) injection site. The reaction occurs at all product sites, irrespective of time, filler type, and number of injections. Approximately 40% of patients develop general flu-like symptoms with fever and/or joint pain [4,15,16]. This reaction can be provoked. The onset of the reaction is most often triggered by another infectious process (sinusitis, urinary tract infection, respiratory tract infection, dental trauma), facial trauma, dental procedures (sandblasting, teeth tartar removal), menstruation, vaccination (including against COVID-19), and drugs, causing an increase in IFN [17,18]. In a study, Owczarczyk and de Boulle presented a case series of ASIA syndrome preceded by DIR (delayed inflammatory reaction) provoked by HAF (hyaluronic acid filler) administration [4]. Activation of the M1 phenotype of macrophages implies their excessive cascade activation, resulting in the development of ASIA syndrome (Figure 4).

Similarly, Montealegre et al. observed 13 patients after buttock augmentation who developed ASIA after injection of biopolymers into the buttocks, referred to as silicone implant incompatibility syndrome. Initial symptoms at the implant site were consistent with a local inflammatory process characterised by pain and continuous or intermittent congestion, even months or years after application of the modelling substance, resembling DIR. More than 50% of the patients had a family history of autoimmunity, and seven patients underwent additional aesthetic procedures. The authors assessed patients according to the ASIA score, which predicts the likelihood of developing this disorder, taking into account age, gender (more common in middle-aged women), personal or familial autoimmune comorbidities, and exposure to other procedures with adjuvant substances (e.g., bioimplants, tattoos, hair dyes, piercings, smoking) [3].

The other important adjuvant present in our patient was multiple tattoos. The dyes used for tattooing contain, among others, aluminium and other metal compounds (nickel, cadmium, chromium, mercury). David et al. described the case of a patient who developed ASIA with symptoms of arthralgia, dryness syndrome, small nerve fibre neuropathy, and post-orthostatic tachycardia syndrome, following a purple tattoo whose dye contains aluminium salts [19]. The presence of aluminium in the ink may explain the mechanism by which tattooing can lead to ASIA syndrome, as observed in the patient described. ASIA syndrome has also been described after exposure to nickel and chromium [19]. However, our patient only had black tattoos, mainly containing amorphous carbon molecules, polycyclic aromatic hydrocarbons, chromium, nickel, cobalt, copper, cadmium, and iron oxides. Unfortunately, no government agency formally regulates tattoo inks, and they may contain hazardous chemicals [20].

The largest analysis of as many as 500 ASIA cases by Watad et al. revealed that autoimmune diseases are significantly more common than autoinflammatory diseases after exposure to vaccine adjuvants, especially for the HBV vaccine (OR 6.72). Among these, UCTD (undifferentiated connective tissue disease) had the highest prevalence, followed by Sjögren’s syndrome and SLE, at 22.6%, 9.8%, and 3.6%, respectively [2]. Unfortunately, neutrophilic skin diseases (e.g., Sweet’s syndrome) were not included in this analysis as autoinflammatory diseases.

On the other hand, HA is a ligand for CD44 receptors and has been linked to the pathogenesis of nephritis in SLE patients. Suarez-Fueyo et al. showed that administration of an HA synthesis inhibitor (hymecromone) to lupus-prone mice dramatically inhibited the pathology associated with the disease. They observed a reduction in tissue damage and a decrease in the number of intrarenal lymphoid cells, including CD3+CD4-CD8- double-negative T cells, which are involved in the pathogenesis of SLE. Furthermore, exposure of human peripheral blood mononuclear cells to HA (hyaluronic acid) in vitro increased the generation of CD3+CD4-CD8- T cells [21].

Unfortunately, to date, neutrophilic skin disease (Sweet’s syndrome) has not been described as a consequence of ASIA. Admittedly, it is a disorder associated with hyperactivation of normal neutrophils and activation of the inflammasome. Neutrophils release cytokines to promote the recruitment of additional innate and adaptive immune cells and may also be stimulated in ASIA syndrome, with stimulation of anti-neutrophil cytoplasmic antibody production [11]. In addition, it is a condition observed in numerous disorders associated with infections, vaccinations, inflammatory disorders, and even pregnancy. Inflammatory conditions such as inflammatory bowel disease (IBD), systemic lupus erythematosus (SLE), and other rheumatological diseases and malignancies are associated with the development of classic Sweet’s syndrome [22,23].

It is important to note that risk factors for the development of ASIA include female sex, middle age, exposure to adjuvants (bioimplants, tattoos, hair dyes, piercings), smoking, personal or family history of autoimmune co-morbidities, and history of vaccine reactions [3,24].

Up to date, only one similar case was described in 2022 by Mir et al. [25]. This is a case of a 37-year-old woman who developed severe systemic lupus erythematosus as a consequence of ASIA syndrome induced by the implantation of silicone breasts. In this case, the lupus involved mucocutaneous and musculoskeletal systems. The patient also presented haematological symptoms and was diagnosed with lupus nephritis and neuropsychiatric lupus [25].

## 4. Limitations

Even though our patient developed symptoms of SLE in temporal coincidence with exposure to adjuvants and the onset of ASIA syndrome, we cannot exclude the possibility of the primary SLE in this case. Especially taking into account the positive family history in rheumatic diseases, which can provide genetic predisposition to the development of SLE. On the other hand, it is well known that, in addition to genetic predisposition, environmental factors are also required for the induction of SLE. Thus, in our case, it is very probable that SLE was induced by the adjuvants, which also induced ASIA.

## 5. Conclusions

Early diagnosis requires a multidisciplinary approach and may require immunosuppressive treatment in specific cases. Unfortunately, ASIA syndrome after bioimplantation is still underdiagnosed, probably due to ignorance or diagnostic difficulties, as symptoms are uncharacteristic and there is no immunological marker for this syndrome.

In addition, it can develop several years after the procedure, and it is difficult for both patient and physician to become aware of the connection.

## Figures and Tables

**Table 1:** Major and minor criteria of ASIA. The criteria met by the patient were highlighted by text underlining.

Major Criteria	Minor Criteria
Exposure to the adjuvants prior to clinical manifestation Muscle pain, inflammation of the muscles, or muscle weakness	Appearance of autoantibodies or antibodies targeting a given adjuvant or RF, ANCA, ANA, anti-Sm, anti-RNP, anti-SS-A/Ro, anti-SSB, anti-thyroid antibodies
Joint pain and/or arthritis	Other clinical signs (e.g., irritable bowel)
Chronic fatigue, sleep disturbances	Specific HLA (HLA DRB1, HLA DQB1)
Neurological symptoms (especially related to demyelination)	Development of autoimmune diseases (SLE, MS, Sjögren’s syndrome, autoimmune thyroiditis, autoimmune hepatitis, etc.)
Cognitive impairment, memory loss	
Fever Dry mouth	
Removal of the causative agent brings about improvement	
Typical biopsy of the affected organs	

## Data Availability

The analysed datasets generated during the study are available from the corresponding author on reasonable request.
